# ﻿Taxonomic studies on the genus *Sanicula* (Apiaceae) from China (IV): Clarification of morphology and distribution of *S. rugulosa* and *S. subgiraldii*, with reduction of S. astrantiifolia to a synonym of the former

**DOI:** 10.3897/phytokeys.262.165363

**Published:** 2025-09-12

**Authors:** Hui-Min Li, Jun-Wen Zhu, Xu-Dong Ma, Si-Rong Yi, Chun-Feng Song

**Affiliations:** 1 Jiangsu Key Laboratory for Conservation and Utilization of Plant Resources, Institute of Botany, Jiangsu Province and Chinese Academy of Sciences (Nanjing Botanical Garden Mem. Sun Yat-Sen), Nanjing 210014, Jiangsu, China Jiangsu Province and Chinese Academy of Sciences Nanjing China; 2 Chongqing Three Gorges Medical College, 404120, Wanzhou, Chongqing, China Chongqing Three Gorges Medical College Chongqing China

**Keywords:** Morphology, *
Sanicula
*, taxonomy, Umbelliferae

## Abstract

Through field observations of living plants and the analysis of herbarium collections, including type material, it is demonstrated that *Sanicula rugulosa* can be distinguished from *S. subgiraldii* by several morphological characteristics, including the presence of slightly tripartite basal leaves (vs. deeply ternate leaves), umbellules that typically contain 5–7 flowers (vs. 4–11), 3–8 staminate flowers (vs. 2–3), generally three fertile flowers (vs. 1–3) and a consistently black stylopodium (vs. a whitish or pale green one). Furthermore, *S. astrantiifolia* and *S. hanyuanensis* are not essentially different morphologically from *S. rugulosa* and we, therefore, propose to treat them as synonyms in this study.

## ﻿Introduction

*Sanicula* L. (Apiaceae–Saniculoideae), comprising approximately 47 taxa, is widely distributed from East Asia to North America (Wyk et al. 2013; Yang et al. 2022; Song et al. 2024). China is one of the most important centers of the genus diversity, harboring about 20 species and two varieties (Sheh and Phillippe 2005; Pimenov 2017; Xie et al. 2019; Song et al. 2024). This genus is taxonomically complex due to its considerable morphological variation in rhizomes, leaves, inflorescences and fruits, and is considered relatively primitive within subfam. Saniculoideae Burnett and amongst the more primitive members of Apiaceae (Shan and Constance 1951; Hiroe 1979; Calviño et al. 2008; Li et al. 2023). Traditionally, based on leaf, flower, and fruit characters, Shan and Constance (1951) divided the world *Sanicula* species into five sections —*Tuberculatae*, *Pseudopetagnia*, *Sanicla*, *Sandwicenses* and *Sanicoria* — and indicated that the Chinese taxa belonged to the first three sections. This classification was later accepted by many subsequent authors (Liou 1979; Sheh and Phillippe 2005; Pimenov 2017). In recent years, critical examination of herbarium specimens and field observations has led to substantial taxonomic revision of this genus in China, including the reduction of three species and two varieties to synonymy (Li and Song 2022; Li et al. 2022; Li et al. 2024), as well as the identification of additional unresolved taxonomic issues.

**Figure 1.**
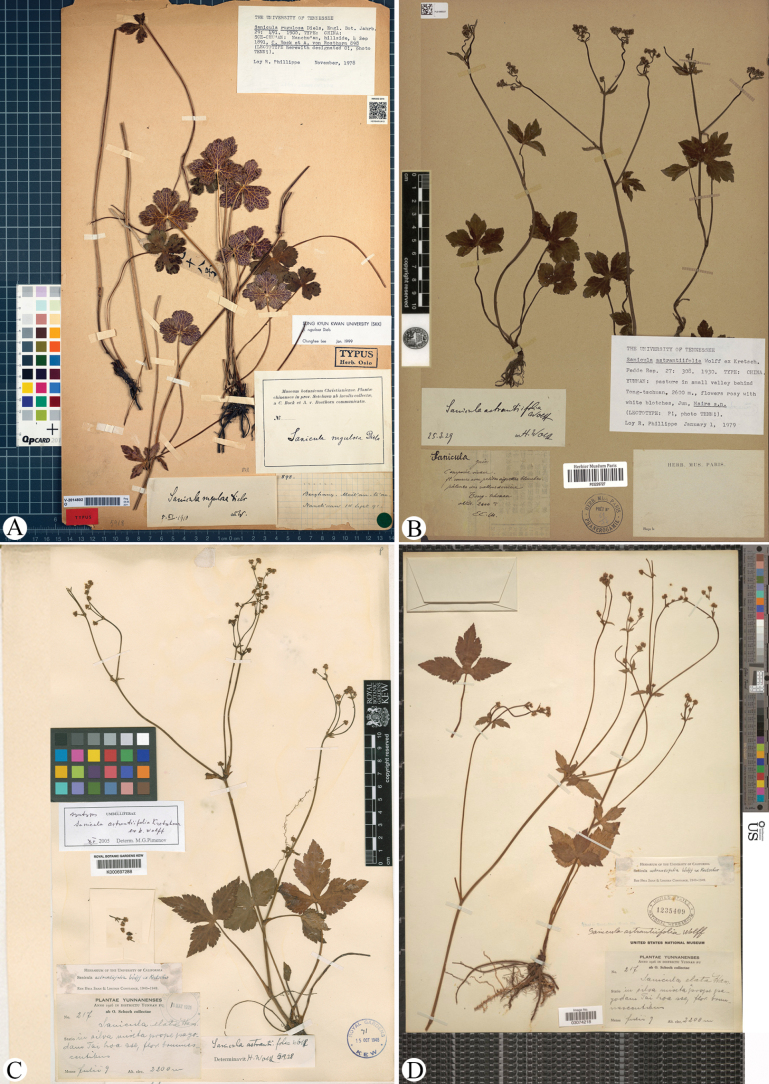
Holotype (A) sheet of *Sanicula rugulosa*, along with the lectotype (B) and syntype (C, D) sheets of *S. astrantiifolia*.

*Sanicula rugulosa* Diels was first described based on a single specimen, *C. Bock & A. von Rosthorn 898* (O-V2014802; Fig. 1A), which was collected from Meitancao (also referred to as Meitan-ts’ao) on Mount Jinfo in Nanchuan County, in southern Chongqing Municipality. In the protologue, Diels (1900) stated that the species was represented solely by a specimen containing fallen fruits. He underscored that the leaf texture and morphology were not congruent with any previously recognized species, noting the presence of firm, papery leaves, a papillose surface above the veins due to impressed venation, and subrugulose, glabrous lower surfaces, with veins displaying a whitish to purplish coloration.

Additionally, Diels (1900) observed that the leaf shape of *S. rugulosa* bore the closest resemblance to that of *S. hacquetioides* Franch., a species endemic to western Yunnan. However, he highlighted notable differences in the morphology of the bracts. Subsequently, Wolff (1913) acknowledged *S. rugulosa* as a valid species, although he remarked that the absence of flowers and mature fruits impeded a definitive evaluation of its taxonomic classification. Based on its overall habit, floral characteristics, branching pattern, and leaf traits, Wolff (1913) proposed its possible relationship with *S. europaea* L.

Since its initial description, *S. rugulosa* has been consistently recognized as a distinct species by various authors, including Hiroe (1979), Liou (1979, 1986), Sheh and Phillippe (2005), and Pimenov (2017). It is noteworthy that the species has been documented only within a restricted geographic range, specifically in Nanchuan, Chongqing, and Bomê, Xizang (Liou 1979, 1986; Sheh and Phillippe 2005; Pimenov 2017).

*Sanicula astrantiifolia* Wolff was initially characterized based on three specimens: *E.E. Maire s.n.* (P03226727; Fig. 1B), *S. Ten 287* (not seen), reported to exhibited red to slightly whitish flowers, and *O. Schoch 217* (K000697288, US03074218; Fig. 1C, D), which displayed brownish flowers. All specimens were collected from Huize County, historically referred to as Tong-tschuan or Dongchuan, within the jurisdiction of Qujing City, Yunnan Province, China. In the protologue, Wolff (1930) did not designate a type for *S. astrantiifolia*, and identified several distinctive morphological traits: the leaves were tripartite to approximately one-fifth of their length; the involucres bracteoles numbered five to seven, were linear-lanceolate, and measured about 2 mm in length; the flowers were subsessile; the calyx teeth exceeded the length of the tube; the petals were oblong, slightly margined, and lobed, with an inflexed apex equal in length to the blade; the stylopodium was thick; the styles were notably long, slender, curved; and the fruits were brown, densely covered with hooked bristles.

Since its original description, *S. astrantiifolia* has been recognized by various authors, including Handel-Mazzetti (1933), Shan (1936), Shan and Constance (1951), Anonymous (1972), Hiroe (1979), Liou (1979, 1986), Wu (1984), Wu (1993), Liu (1997), Sheh and Phillippe (2005) and Pimenov (2017). The species has been documented in south-western Sichuan, southern Xizang, and Yunnan (Sheh and Phillippe 2005; Pimenov 2017).

**Figure 2.**
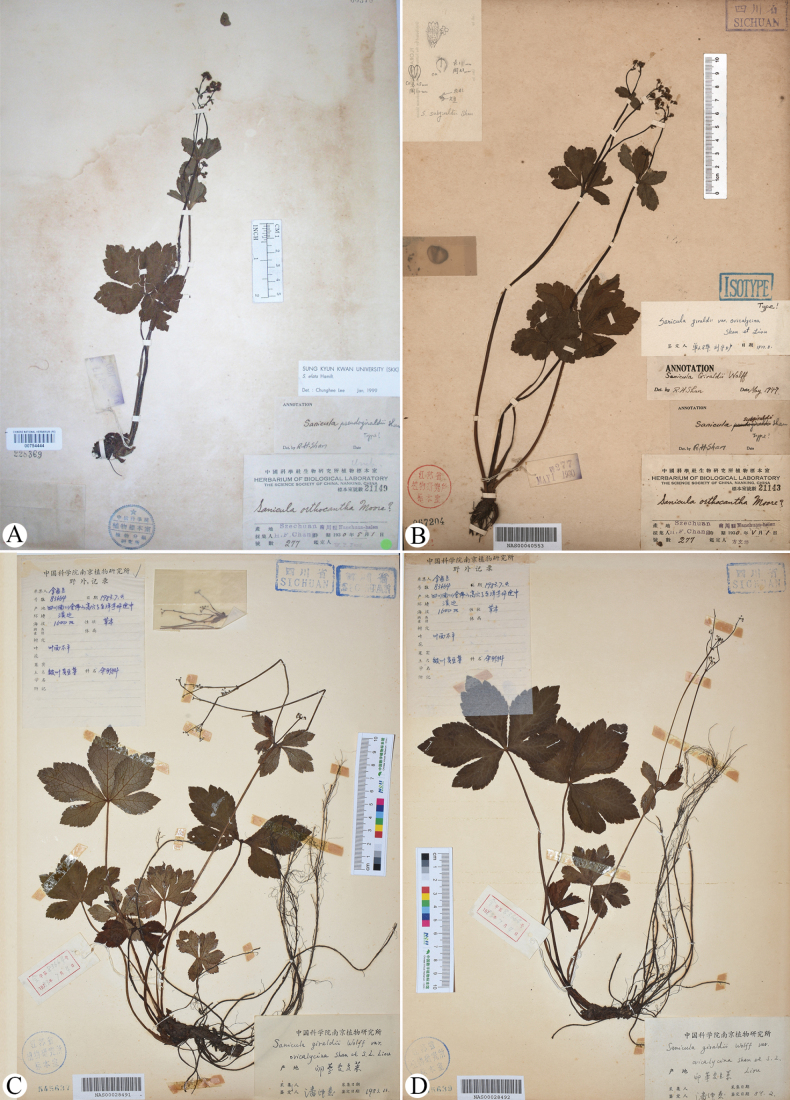
Holotype (A), isotype (B) and additional specimens (C, D) of *Sanicula subgiraldii*.

*Sanicula subgiraldii* Shan was initially described based on a solitary specimen, *H.F. Chang 277* (PE00754444, NAS00040553; Fig. 2A, B), which was collected from Nanchuan, Chongqing, China (not Sichuan, as erroneously noted in some references), with the PE sheet (Fig. 2A) of the collection designated as the holotype (Shan 1943). In the protologue, Shan (1943) observed its morphological resemblance to *S. giraldii* Wolff, particularly in terms of its spiny fruits and generally slender growth form. However, he differentiated *S. subgiraldii* by its smaller primary prophylls, elongated flowering branches, and leaf segments that exhibited a basally obsoletely setulose-crenate morphology.

Later, Liou (1979) acknowledged this entity but classified it as a variety of *S. giraldii*, designating it as S. giraldii var. ovicalycina Shan & Liu. He noted that this variety typically produced one to three fertile flowers per umbellule, featured broadly ovate calyx teeth measuring approximately 1 mm in length and 0.7 mm in width, and yielded relatively larger fruits. However, subsequent scholars expressed differing opinions on this classification. For instance, Sheh and Phillippe (2005) and Pimenov (2017) supported Liou’s varietal designation, while Song et al. (2024) upheld Shan’s original classification at the species level.

This taxon has been documented in both Chongqing and Sichuan (Song et al. 2024). A thorough examination of herbarium specimens and relevant literature concerning the genus *Sanicula* revealed that *S. subgiraldii* has frequently been conflated with *S. rugulosa*, primarily due to inadequate field studies and a limited understanding of its distinguishing characteristics. For example, the specimen *M.L. Sheh* 83664 (NAS00028491, NAS00028492; Fig. 2C, D), collected from Mount Jinfo, Nanchuan, was misidentified by Sheh—one of the authors of the “*Flora of China*” treatment of *Sanicula* — as “皱叶变豆菜”, which referred to *S. rugulosa*, as indicated on the collection label. However, this specimen was subsequently re-identified by Pan in 1983 as S. giraldii var. ovicalycina. This misidentification underscored a broader issue regarding the long-standing lack of clarity and accurate interpretation of the morphological traits associated with *S. rugulosa*.

**Figure 3.**
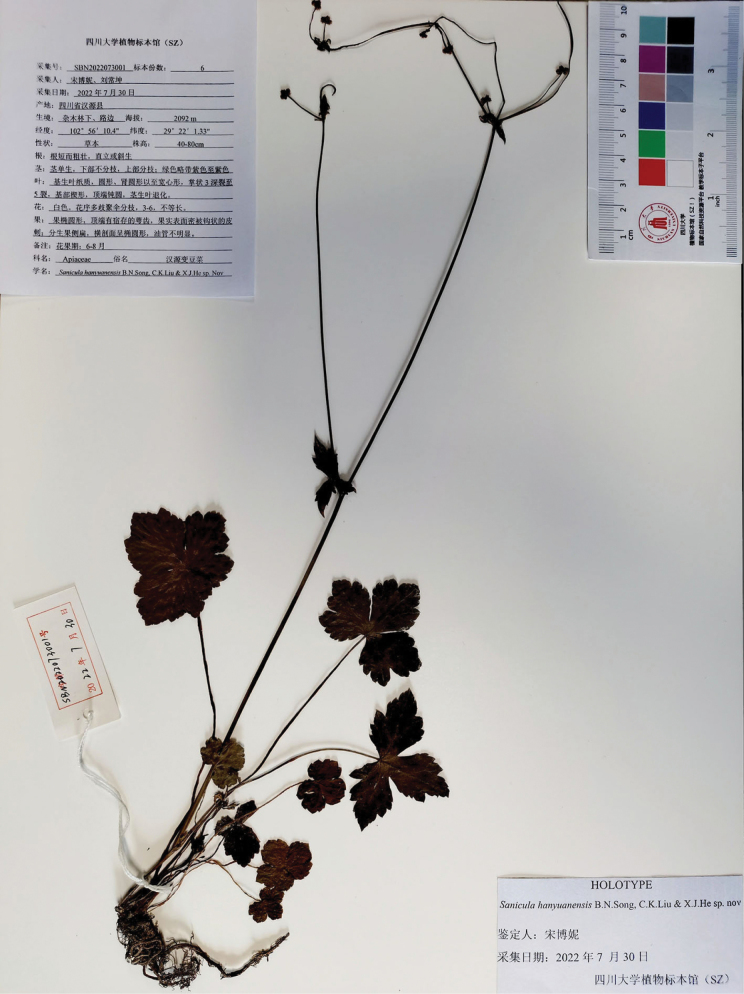
Holotype sheet of *Sanicula hanyuanensis*.

*Sanicula hanyuanensis* B.N. Song, C.K. Liu & X.J. He was characterized based on a single specimen, *B.N. Song & C.K. Liu SBN2022073001* (SZ; Fig. 3), collected from Hanyuan County, Sichuan. In the protologue, Song et al. (2024) described the basal leaves as numerous, with leaf blades exhibiting orbicular, reniform-rounded, or broadly cordate shapes, and being palmately deeply divided into 3–5 segments. The central segment was noted to be broadly obovate, with a shallow 3-lobed distal end, a cuneate base, and an obtuse-rounded apex. The lateral segments were characterized as rhombic-rounded or broadly obovate, also shallowly 3-lobed at the distal end. The inflorescence was identified as pleiochasium-branched, comprising 3–6 unequal branches; the bracts were either small or degraded, while the bracteoles consisted of two opposite, linear-lanceolate structures measuring 0.6–1 cm long, 0.1–0.4 cm wide. Each umbellule contained 4–7 flowers, which included 3–5 staminate flowers and 1–2 fertile flowers; the pedicels were notably shortened, approximately equal in length to the fertile flowers. The calyx teeth were lanceolate, measuring approximately 1 mm long, 0.5 mm wide, and the styles were about 2 mm in length and recurved. The fruits were described as broadly ovate, measuring approximately 4–5 mm long, 3–4 mm wide, and were densely covered with purplish-red uncinate prickles. Song et al. (2024) highlighted that the species could be distinguished by its pleiochasium inflorescence featuring 3–6 unequal branches, small or degraded bracts, and paired opposite linear-lanceolate bracteoles.

The aim of this study was to elucidate the morphological variation of *Sanicula rugulosa*, to determine the distinctions between *S. subgiraldii* and *S. rugulosa*, and to clarify the identity of *S. astrantiifolia* and *S. hanyuanensis* based on observations of herbarium specimens (including type material) and living plants in the field.

## ﻿Materials and methods

To perform morphological comparisons, we conducted a thorough analysis of specimens and high-resolution images of relevant *Sanicula* taxa obtained from various herbaria, including CDBI, E, GZTM, HIB, HITBC, HNWP, K, KUN, L, LBG, NAS, NY, O, P, PE, PEY, SM, SWFC, SZ, US and WUK. Furthermore, field observations were carried out across eight populations situated in the provinces of Chongqing, Sichuan, and Yunnan (Table 1). Of particular note are four populations: two from Mount Jinfo in Nanchuan County, southern Chongqing, designated as the type localities for *S. rugulosa* and *S. subgiraldii*, respectively; one from Huize County in Qujing City, northeastern Yunnan, recognized as the type locality for *S. astrantiifolia*; and one from Hanyuan County, Sichuan, which served as the type locality for *S. hanyuanensis*. The morphological comparisons presented in this study were based on a detailed examination of both herbarium specimens and fresh materials collected during fieldwork.

**Table 1.** The field collection information of seven populations of *Sanicula rugulosa* and one population of *S. subgiraldii.*

Taxon	Voucher	Locality	Figure
*Sanicula rugulosa*	*X.D. Ma 310* (NAS)	Yunnan, Lijiang City	–
	*X.D. Ma 315* (NAS)	Yunnan, Shangri-La City, Haba Snow Mountain	–
	*H.M. Li, J.W. Zhu & C.F. Song 1547* (NAS)	Sichuan, Hanyuan County, Malie Township	Fig. 8
	*S.R. Yi LHM3005* (NAS)	Chongqing, Nanchuan County, Mount Jinfo	Fig. 5
	*H.M. Li & J.W. Zhu 3002* (NAS)	Yunnan, Qujing City, Huize County, Fengdou Village	Fig. 6
	*H.M. Li & J.W. Zhu 3004* (NAS)	Yunnan, Kunming City, Xishan District, Tuanjie Township	–
	*H.M. Li & J.W. Zhu 3003* (NAS)	Yunnan, Qujing City, Huize County, Hongyan Village	–
*S. subgiraldii*	*H.M. Li, Y.S. Zhang & X. Zhang 1143* (NAS)	Chongqing, Nanchuan County, Mount Jinfo	Fig. 9

Numerical analyses of leaf incision and apical acuteness of the central lobe were conducted based on measurements from herbarium specimens of *Sanicula rugulosa* collected from 20 populations (Fig. 7) in Chongqing, Guizhou, Sichuan, Xizang, and Yunnan. ImageJ was used to perform quantitative measurements, and R was employed to generate visualizations.

## ﻿Results and discussion

**Figure 4.**
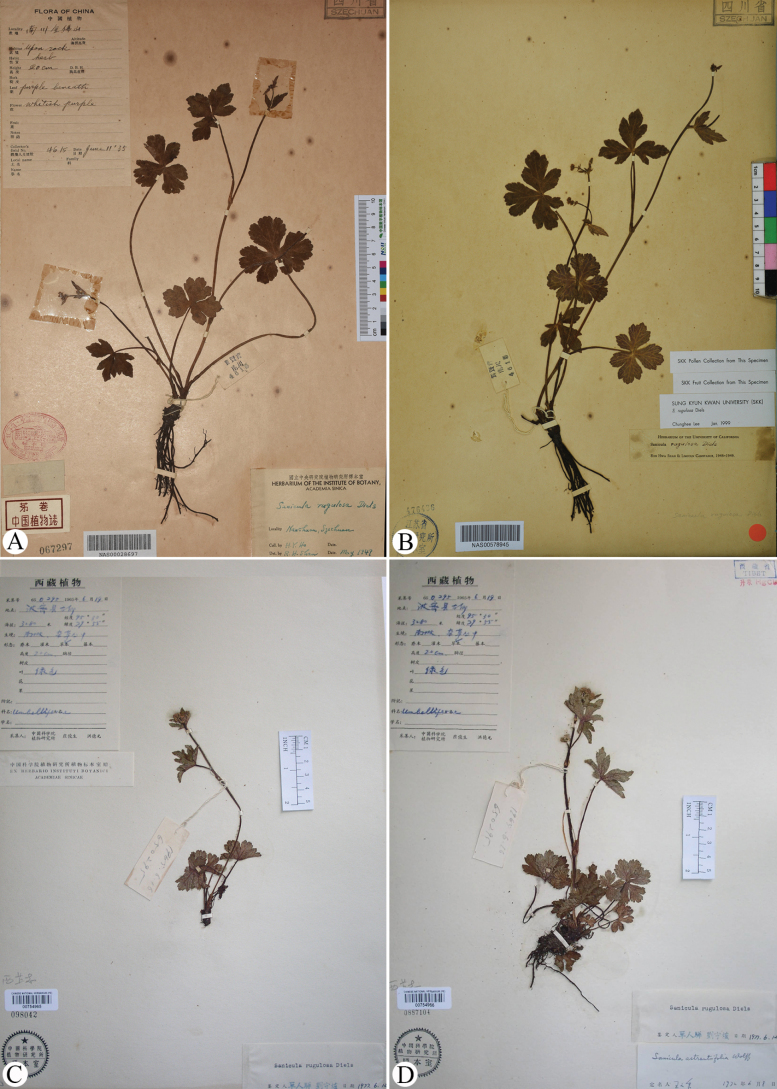
Specimens of *Sanicula rugulosa*. A, B. Chongqing, Nanchuan County, Mount Jinfo, *H.Y. Ho 4615* (NAS); C, D. Xizang, Bomê County, *J.S. Ying & D.Y. Hong 650295* (PE).

Following the original characterization of *Sanicula rugulosa*, Diels (1900) and Wolff (1913) acknowledged the species despite the absence of floral and mature fruit records. Notably, due to the lack of floral characteristics in the type material, Hiroe (1979) referenced a representative specimen, *H.Y. Ho 4615* (NAS00028697, NAS00578945; Fig. 4A, B; correcting Hiroe’s previous misidentification “4616”) from the type locality in Mount Jinfo, Nanchuan County, Chongqing. This specimen documented umbels with 3–5 rays (rays measuring 0.2–2 cm), eight linear involucel bractlets, and umbellules comprising 5–7 flowers, including 2–3 short-pedicellate staminate flowers (with narrowly lanceolate calyx lobes approximately 1 mm in length and white petals) and approximately three subsessile fertile flowers, whose calyx lobes enlarged in fruit and whose styles were recurved and slightly exceed the calyx. The fruits exhibited laterally compressed mericarps with flat seed faces and inconspicuous vittae. Subsequently, Liou (1979, 1986) accepted the species and documented intraspecific variation in petal color (ranging from white to pale blue) based on specimens, *J.S. Ying & D.Y. Hong 650295* (PE00754965, PE00754966; Fig. 4C, D), collected from Bomê County, Xizang. These specimens (Fig. 4C, D) were identified by different scholars as either *S. rugulosa* or *S. astrantiifolia* within the taxonomic complex.

**Figure 5.**
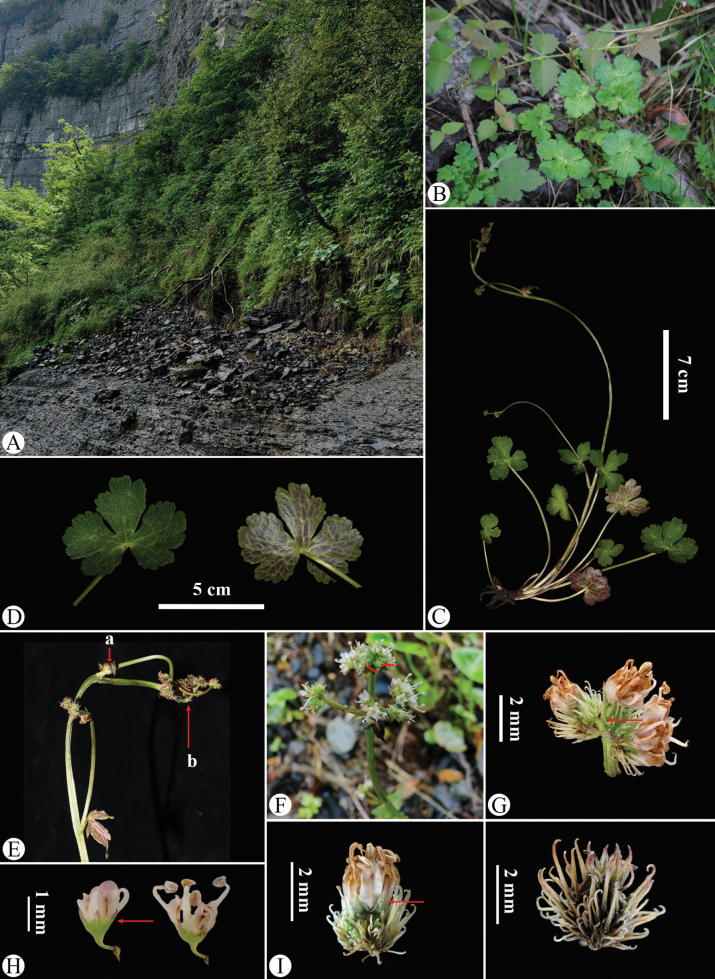
*Sanicula rugulosa* in the wild (China, Chongqing, Nanchuan County, Mount Jinfo, the type locality of *S. rugulosa*). A. Habitat; B. Habitat and habit during the early flowering period; C. Habit during the mature flowering and fruiting period; D. Leaf (left: adaxial surface; right: abaxial surface); E. Portion of the inflorescence during the fruiting period, with the arrow (a) indicating the cauline leaf and (b) indicating the involucrate bract; F. Portion of the inflorescence, with the arrow indicating the reduced and non-reduced stylopodium in pale green; G. Umbellule (side view), with the arrow indicating the involucellate bracteoles; H. Staminate flower (side view), with the arrow indicating the calyx teeth; I. Fertile flower with fruit, with the arrow indicating the calyx teeth; J. Mericarps. Photographed by Hui-Min Li and Si-Rong Yi.

**Figure 6.**
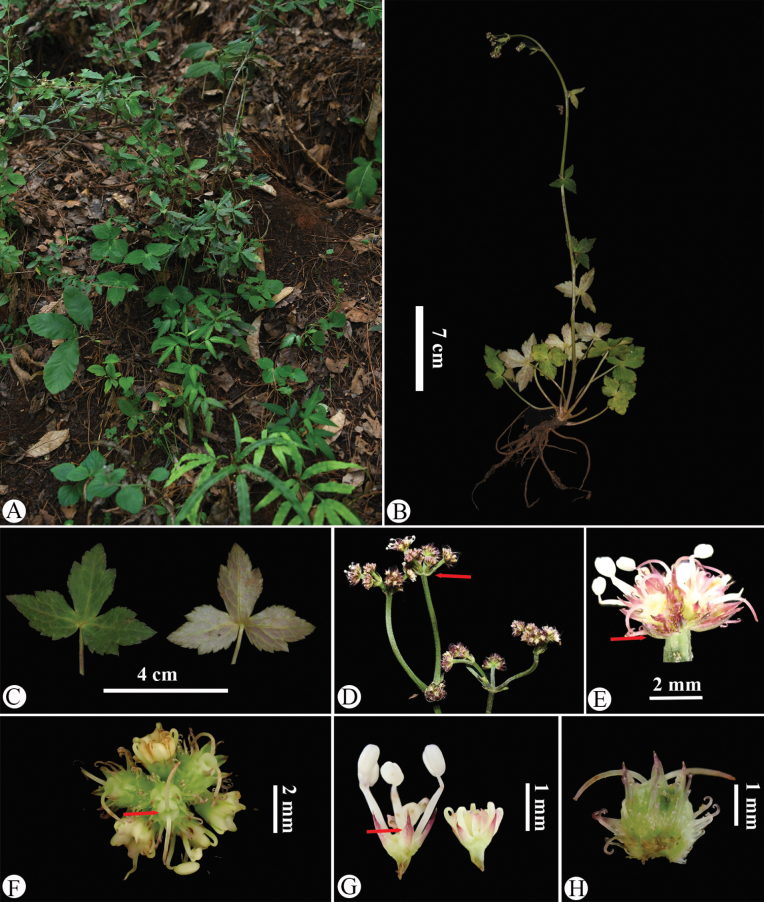
*Sanicula rugulosa* in the wild (China, Yunnan, Huize County, the type locality of *S. astrantiifolia*). A. Habitat; B. Habit; C. Leaf (left: adaxial surface; right: abaxial surface); D. Portion of the inflorescence during the early fruiting period, with the arrow indicating the involucrate bract; E. Umbellule (side view), with the arrow (a) indicating the involucellate bracteoles; F. Umbellule (top view), with the arrow indicating the stylopodium in pale green; G. Staminate flower (side view), with the arrow indicating the calyx teeth; H. Mericarps. Photographed by Hui-Min Li.

Building upon these historical observations, our field investigations across seven populations, including type localities in Chongqing, Sichuan, and Yunnan, revealed consistent morphological characteristics. Specifically, at the type locality of *S. rugulosa* (Mount Jinfo, Nanchuan County, Chongqing), the plants displayed orbicular to cordate basal leaves (measuring 1.6–3.5 × 3.5–5.5 cm), palmately 3-parted of 3/4–9/10 of the way to the base (Fig. 5C), the terminal inflorescences were characterized by approximately 3-radiate umbels (rays measuring 0.5–2 cm), the reduced and non-reduced stylopodium were pale green (Fig. 5F) and fruits were characterized by uncinate bristles (Fig. 5I). Similarly, observations at the type locality of *S. astrantiifolia* (Huize County, Yunnan) revealed strikingly analogous characteristics: broadly cordate leaves that were 3-parted of 7/10–9/10 of the way to the base (Fig. 6C), inflorescence terminals featuring approximately 3-radiate umbels with rays measuring 0.5–1.5 cm (Fig. 6D), pale green reduced and non-reduced stylopodia (Fig. 6F), and fruits characterized by uncinate bristles (Fig. 6H).

**Figure 7.**
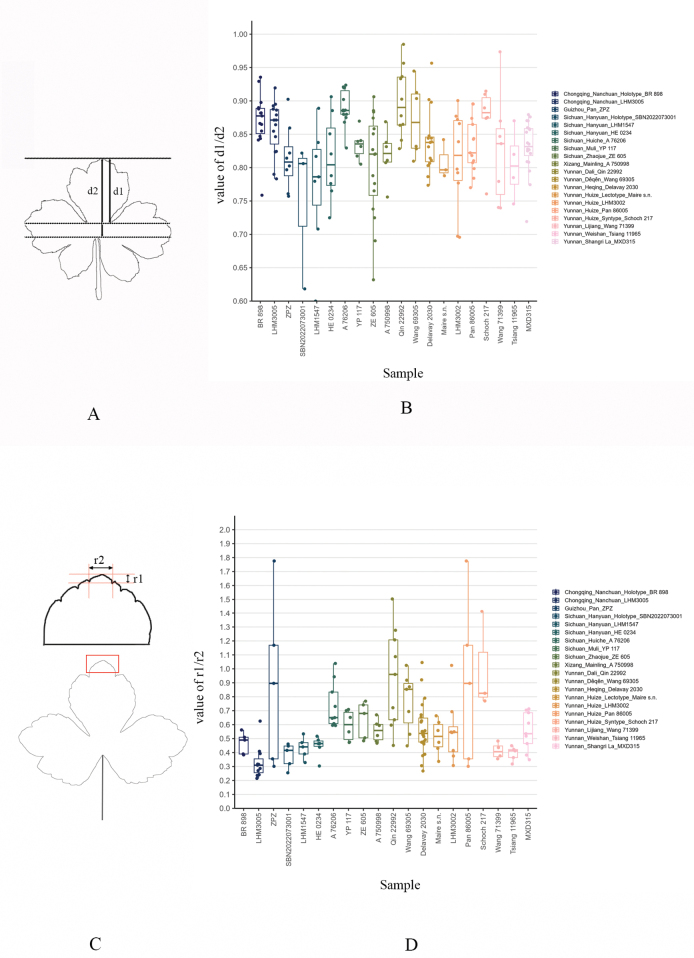
Variation in basal leaf morphology ratios (d1/d2, r1/r2) across 20 populations of *Sanicula rugulosa*. A. Line drawing of a basal leaf showing measurement positions for d1 and d2 (quantified in Suppl. material 1); B. Boxplots of d1/d2 ratios per population. The filled circles indicate raw measurements, while the boxes illustrate the interquartile ranges (IQR, Q1-Q3). The horizontal lines within the boxes denote the median values. The whiskers extend to ± 1.5 times the IQR from the quartiles and any outliers beyond the whiskers are displayed individually. The presence of continuous intraspecific variation is demonstrated by the overlapping ranges of values and the clinal transitions observed amongst the populations; C. Line drawing of a leaf lobe showing measurement positions for r1 and r2 (quantified in Suppl. material 2); D. Boxplots of r1/r2 ratios per population (elements identical to B).

Historically, however, discrepancies have persisted. Shan (1936) described *S. astrantiifolia* as possessing “trifoliate leaves not divided to the base” and “horizontal lateral segments”, while Shan and Constance (1951) highlighted its “constantly tripartite leaves with unlobed segments” to differentiate it from *S. rugulosa*. Similarly, Liou (1986) acknowledged the extreme similarity but cited the depth of leaf division (2/3–3/4 vs. 4/5–5/6 of the way to the base) as a distinguishing feature of *S. astrantiifolia*. In contrast to these assertions, our morphometric data (Suppl. materials 1, 2; Fig. 7) and population-level sampling demonstrate continuous variation in leaf architecture (including leaf incision and apical acuteness of the central lobe), thereby undermining all previous diagnostic boundaries.

**Figure 8.**
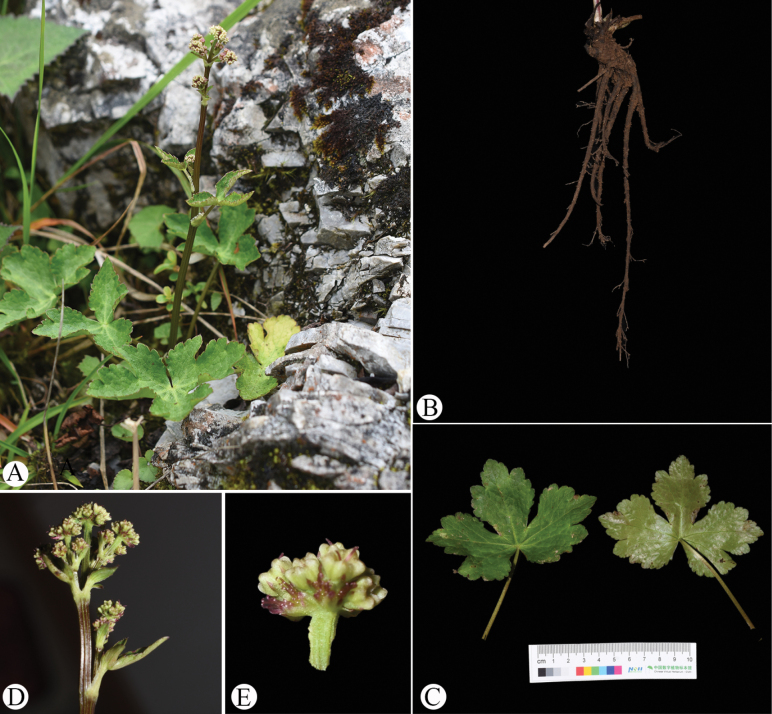
*Sanicula hanyuanensis* in the wild (China, Sichuan, Hanyuan County, the type locality of *S. hanyuanensis*). A. Habitat and habit; B. Rhizome; C. Leaf (left: adaxial surface; right: abaxial surface); D. Portion of the inflorescence during early flowering and fruiting period; E. Umbellule (side view).

An analysis of the type material of *Sanicula hanyuanensis* (Fig. 3) has revealed several key morphological characteristics, including a short, robust, fascicled, fleshy rhizome; basal leaves that were orbicular, reniform or broadly cordate in shape (measuring 1.2–3.3 × 2–6 cm) and palmately 3-parted to varying degrees (3/5–9/10 of the way to the base); terminal inflorescences featuring approximately 2–3-radiate umbels (with rays measuring 0.6–1.5 cm); and fruits adorned with uncinate bristles (Fig. 4). These morphological traits were further validated through field observations of living specimens (Fig. 8) and collections made at the type locality in Hanyuan County, Sichuan. Notably, the timing of the fieldwork coincided with the early flowering and fruiting phases of the plants, which consistently exhibited a short, slightly fleshy rhizome; basal leaves that ranged from orbicular to cordate (1.5–7 × 3–9 cm) and were palmately 3-parted (7/10–9/10 of the way to the base) (Fig. 8C); terminal inflorescences with approximately 3-radiate umbels (with rays measuring approximately 0.7 cm); and fruits characterized by uncinate bristles (Fig. 8E). While Song et al. (2024) highlighted the presence of paired, opposite, linear-lanceolate bracteoles as distinguishing features, it is important to note that this structure is more accurately described as involucellate bracts, which typically number between one and three and exhibit a 3-lobed or lanceolate form. Therefore, the morphological continuum observed across all examined specimens indicates that *S. hanyuanensis* cannot be distinguished from *S. rugulosa*, thereby supporting the classification of these taxa as a single species.

**Figure 9.**
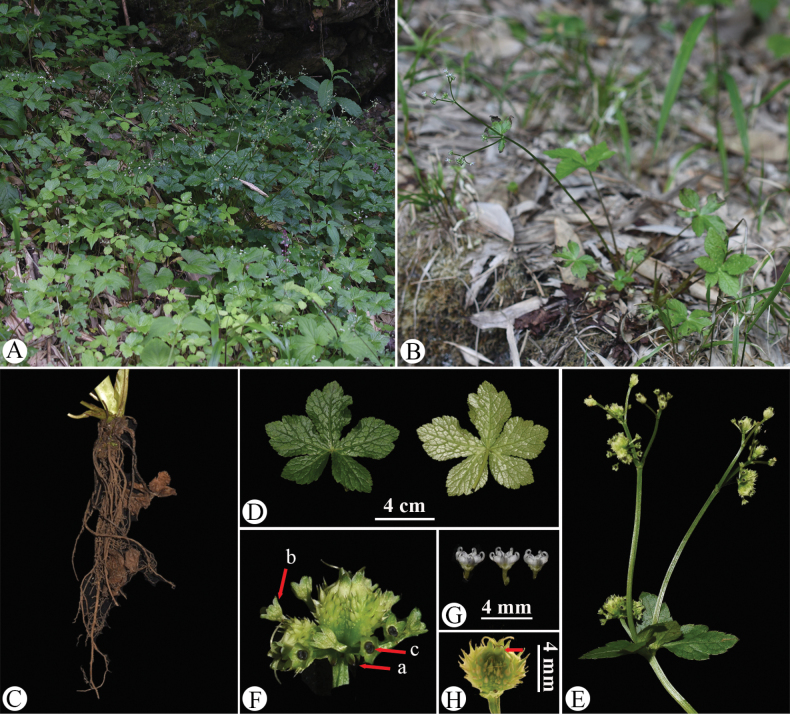
*Sanicula subgiraldii* in the wild (China, Chongqing, Nanchuan County, Mount Jinfo, the type locality of *S. subgiraldii*). A. Habitat; B. Habit; C. Rhizome; D. Leaf (left: adaxial surface; right: abaxial surface); E. Portion of the inflorescence during fruiting period; F. Umbellule (side view), with the arrow (a) indicating the involucellate bracteoles, (b) indicating the calyx teeth and (c) indicating the reduced stylopodium black. Photographed by Hui-Min Li.

In addition, to address the persistent taxonomic ambiguities, our reassessment of *Sanicula subgiraldii*—conducted through an extensive analysis of herbarium specimens, including type material, and field observations at its type locality, Mount Jinfo, Chongqing (Table 2; Fig. 9)—has elucidated stable, non-overlapping distinctions from *S. rugulosa*. The characteristics that define *S. subgiraldii* include: (1) deeply ternate basal leaves divided into three distinct leaflets (Fig. 9D); (2) larger umbellules comprising 4–11 flowers (in contrast to 5–7 in *S. rugulosa*); (3) a higher number of staminate flowers (3–8 vs. 2–3); (4) a reduced number of fertile flowers (1–3 vs. typically 3); and, importantly, (5) a consistently black stylopodium, both reduced and non-reduced (Fig. 9F), which stands in stark contrast to the whitish or pale green stylopodium observed in *S. rugulosa*. This suite of characters provides unambiguous criteria for delimiting *S. subgiraldii* from *S. rugulosa*. As a result, the mixed identification of specimen *M.L. Sheh 83664* (NAS00028491, NAS00028492; Fig. 2C, D) was definitively resolved, confirming its assignment to *S. subgiraldii.*

**Table 2.** Morphological comparisons between *Sanicula rugulosa* and *S. subgiraldii.*

Morphological character	S. subgiraldii	S. rugulosa
**Roots**	rhizome, tuberous and thickened	rhizome, short and stout, roots fascicled, fleshy, slightly fibrous
**Stems**	stems 1–2, slender and ascending, 15–50 cm tall	stems usually solitary, erect, branched distally, 7–52(–90) cm tall
**Basal leaves**	1–12; petioles much longer than the blade, basally membranously sheathed; blade 2.5–6 cm long, 4–15 cm wide, ternate, median segments obovate, cuneate at base, blunt or acuminate at apex, obscurely trilobed; lateral segments oblique, rhomboid-ovate, divided up to 2/3 of the way to the base, with intermediate lobes obovate, all segments with margins setulose-crenate to serrate, lowest teeth nearly obsolete	2–8, long petiolate; petioles 5–20 cm long; basally membranously sheathed; blade 2–5 cm long, 4–8 cm wide, palmately 3-parted to nearly 3/4–9/10 of the way to the base; abaxially pale green, occasionally purplish-red; central segment broadly obovate, distally shallowly 3-lobed, base cuneate, apex obtuse-rounded; lateral segments oblique-reniform, rhombic-rounded or ovate-lanceolate, often 2-lobed, with margins crenate to irregularly bi-spinulose-serrate
**Cauline leaves**	ternate, smaller than, but similar to basal leaves, 2–4 × 2–7 cm, less dissected	3-parted nearly 4/5 of the way to the base, segments obovate to ovate-lanceolate, 2–5 × 2–9 cm
**Inflorescence**	1 to several branched, ca. 8 cm long	2–3-dichotomously branched, 2–12 cm long
**Involucrate bracts**	ca. 2, broadly ovate	1–3, either deeply 3-lobed or lanceolate
**Rays of umbels**	terminal umbels 2–3-rayed; rays 3–10 mm long	terminal umbels 3-rayed; rays ca. 2–25 mm long
**Involucellate bracteoles**	ca. 9, ovate	7–10, lanceolate to ovate-lanceolate
**Umbellules**	4–17-flowered, calyx teeth ovate, obtuse, submucronate, 0.5–0.7 mm long	5–7-flowered, calyx teeth narrowly lanceolate, acute, ca. 1 mm long
**Staminate flowers**	3–14 per umbellule; pedicels ca. 2 mm; petals white; reduced stylopodium black	2–3 per umbellule; pedicels ca. 1 mm long; petals white to purple blue; reduced stylopodium whitish to pale green
**Fertile flowers**	1–3 per umbellule; styles ca. 2 mm, recurved; stylopodium black	3 per umbellule; styles ca. 1.5 mm, recurved; stylopodium whitish to pale green
**Mericarps**	orbicular-ovoid, 3–5 × 3.3–5 mm, densely covered with hooked prickles	obovate to ellipsoid, 1.5–2.2 × 2–2.4 mm, densely covered with uncinate bristles, yellow or purplish-red at maturity
**Vittae**	obscure	obscure

Moreover, *S. subgiraldii* is clearly distinguished from *S. giraldii* by a suite of morphological characters, particularly in the leaves, calyx, stylopodium, and fruits. The leaves exhibit prominently raised veins and a rugose surface (vs. veins not prominent and surface smooth); the calyx teeth are markedly smaller, measuring 0.5–0.7 mm in length and 0.3–0.5 mm in width (vs. 1–1.2 mm in length and 0.8–1 mm in width); and, most notably, the stylopodium is consistently black (vs. green). The fruits also differ, being 1–3 in number and 3–5 mm long × 3.3–5 mm wide (vs. consistently 3 in number and 1.4–3.3 mm long × 1.4–2.3 mm wide). The stability of these diagnostic traits across all examined specimens, including type materials, strongly supports the recognition of *S. subgiraldii* at the species rank, as originally proposed by Shan (1943) and reaffirmed by Song et al. (2024), rather than its treatment as a variety of *S. giraldii*.

Consequently, we arrived at two primary taxonomic conclusions: (1) *Sanicula astrantiifolia* and *S. hanyuanensis* are synonymized under *S. rugulosa*, falling within the morphological variation observed in the latter; and (2) *S. subgiraldii* is affirmed in its species status, being distinctly identifiable by its deeply ternate leaves, floral arrangement, and the presence of a uniquely black stylopodium—findings that align with the taxonomic framework proposed by Song et al. (2024).

### ﻿Taxonomic treatments

#### ﻿ Sanicula rugulosa

Taxon classificationPlantaeApialesApiaceae

Diels, Bot. Jahrb. Syst. 29(3–4): 491 (1900).

B9A16B2B-37DF-5B9F-B066-3030BCDA42A1

Figs 1, 3, 4, 5, 6, 8

= Sanicula astrantiifolia H. Wolff ex Kretschmer, Repert. Spec. Nov. Regni Veg. 27: 308 (1930), syn. nov. Type: CHINA. Yunnan, Qujing, Huize (historically referred to as Tong-tschuan or Dongchuan), alt. 2600 m, *E.E. Maire s.n.* [lectotype: P03226727! designated by Pimenov (2017)].
= Sanicula hanyuanensis B.N. Song, C.K. Liu & X.J. He, Frontiers Pl. Sci. (Online journal) 15–1351023: 13 (2024), syn. nov. Type: CHINA. Sichuan, Hanyuan, under the mixed forest or roadsides; 29°22'1.33"N, 102°56'10.4"E; alt. 2092 m, 7 July 2022, *B.N. Song & C.K. Liu SBN2022073001* (holotype: SZ!).

##### Type.

China • Chongqing, Nanchuan, Mount Jinfo, Meitancao (= Meit’an ts’ao), 4 September 1891, *C. Bock & A. von Rosthorn 898* (holotype: O-V2014802!). Fig. 1A

##### Description.

Perennial. Rhizome, short and stout, roots fascicled, fleshy, somewhat fibrous. Stems erect, branched above, 7–52(–90) cm tall. Basal leaves 2–8, long petiolate; petioles 5–20 cm long; blade glabrous adaxially and abaxially, occasionally purplish-red on the back, 2–5 cm long, 4–8 cm wide, palmately 3-parted to nearly 3/4–9/10 of the way to the base; abaxially pale green, occasionally purplish-red; crenate, serrate or irregularly doubly spinose-serrate; central segment broadly obovate, distally shallowly 3-lobed, base cuneate, apex obtuse-rounded; lateral segments oblique-reniform, rhombic-rounded or ovate-lanceolate, often 2-lobed. Cauline leaves reniform-rounded, 3-parted nearly 4/5 of the way to the base, segments obovate to ovate-lanceolate. Inflorescence 2–3-dichotomously branched; involucrate bract 1–3, 3 deeply lobed or lanceolate, acuminate; terminal umbels around 3 radiate, rays 0.2–2.5 cm long; involucellate bracteoles 7–10, lanceolate to lanceolate ovate, ca. 1–2 mm long. Umbellules 5–7-flowered, staminate flowers 2–3 per umbellule, pedicels around 1 mm long, petals mainly white to purple blue, reduced stylopodium whitish or pale green. Fertile flowers usually 3 per umbellule, sessile or short-pedicellate; calyx teeth narrowly lanceolate, acute, ca. 1 mm long; styles ca. 1.5 mm long, recurved, stylopodium whitish or pale green. Mericarps obovate, subglobose or ellipsoid, 1.5–2.2 mm long, 2–2.4 mm wide, densely covered with uncinate bristles when mature, bristles yellow or purple-reddish. Vittae obscure.

##### Distribution.

**Figure 10.**
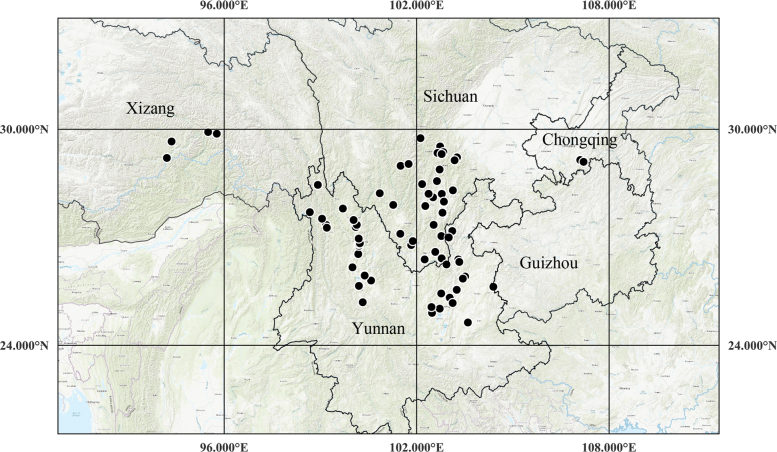
The distribution of *Sanicula rugulosa* (black circle).

*Sanicula rugulosa* is widely distributed in China (Chongqing, Guizhou, Sichuan, Xizang and Yunnan) (Fig. 10).

##### Habitat.

The species grows on mountain slopes under forest or along ravine streams near rocks or at forest margins at elevations of 1400–3400 m above sea level.

##### Phenology.

Flowering from later May to July, fruiting from June to September.

##### Etymology.

The epithet *rugulosa* is derived from the Latin term referring to the abaxial leaves sub-rugulose to rugulose.

##### Additional specimens examined.

China • **Chongqing Municipality**, Nanchuan County, 11 June 1935, *H.Y. Ho 4615* (NAS); Nanchuan County, 30 June 1935, *Anonymous 5268* (NAS); Nanchuan County, Mount Jinfo, 29°6'27.0019"N, 107°12'13.60"E, alt. 2086 m, 19 June 2025, *S.R. Yi LHM3005* (NAS). • **Guizhou Province**, Pan County, Huopu town, 25°39'18.1"N, 104°23'28.8"E, alt. 2026.8 m, 10 November 2015, *P.Z. Zhai 520222151110005LY* (GZTM). • **Sichuan Province**, Butuo County, alt. 2410 m, 24 August 1959, *Sichuan Econ. Plant Exped. 5823* (KUN, PE, SM); Ebian Yi Autonomous County, Laoyingzui, alt. 2100 m, 13 July 1930, *W.P. Fang 7349* (NAS); Ebian Yi Autonomous County, Xinchang Township, alt. 1400 m, 25 June 1979, *Ebian Exped. 338* (SM); Ganluo County, Puchang Town, *Anonymous 715* (SM); Hanyuan County, Malie Township, alt. 1700 m, 24 May 1978, *Hanyuan Exped. 0234* (SM); Hanyuan County, Malie Township, alt. 2200 m, 11 June 1978, *Hanyuan Exped. 0279* (SM); • Hanyuan County, Malie Township, Jianzhuping, 29°22'20.856"N, 102°49'56.28"E, alt. 2316.32 m, 3 June 2024, *J.W. Zhu, H.M. Li & C.F. Song 1547* (NAS); Hanyuan County, Wanli Township, alt. 1300 m, 12 September 1978, *Hanyuan Exped. 0983* (SM); Hanyuan County, Yongli Township, 1972, *Yajiang Exped. 72–76* (SM); Huidong County, alt. 1800 m, 29 May 1978, *Huidong Exped. 0019* (SM); Huidong County, Tangtang Town, alt. 2500 m, 30 May 1976, *Anonymous 76206* (NAS); Huili County, Taiping Town, 25 June 1978, *Anonymous 53* (SM); Jiulong County, alt. 2000 m, 3 July 1979, *Anonymous 121* (SM); Jiulong County, Wulaxi Township, alt. 2200 m, 18 June 1974, *Q.Q. Wang 4572* (CDBI, NAS); Luding County, Xinxing Township, alt. 2250 m, 13 June 1983, *Sichuan Plant Exped. 31115* (CDBI); Meigu County, Shangang Village, alt. 1900 m, 26 June 1979, *Meigu Exped. 0294* (SM); Mianning County, Senrong Town, alt. 1800 m, 19 July 1979, *Mianning Exped. 858* (SM); Miyi County, Baipo Mountain, alt. 2500 m, 10 July 1983, *Qinghai-Tibet Plateau Exped. 11916* (KUN); Miyi County, Malong Township, alt. 2500 m, 1 August 1958, *S.Y. Chen, Z. He, M.F. Zhong 11061* (NAS); Miyi County, Puwei Town, 23 June 1958, *S.Y. Chen, Z. He, M.F. Zhong 10376* (NAS); Muli County, Southern slope of Kangwuliangzi, alt. 2500 m, 14 October 1982, *F.T. Pu & K. Yao 117* (NAS); Muli County, Tangkur Town, alt. 2600 m, 24 July 1978, *Muli Exped. 0515* (SM); Ningnan County, Luoge Township, alt. 2080 m, 14 June 1978, *Ningnan Exped. 0158* (SM); Puge County, alt. 2500 m, 8 August 1979, *Puge Exped. 516* (SM); Xichang City, 1 August 1958, *Anonymous 19011* (SM); Xide County, Mishi Town, alt. 2100 m, 27 July 1979, *Anonymous 626* (SM); Xide County, Zeyue Village, 14 June 1979, *Anonymous 413* (SM); Yanbian County, Guosheng Town, *Anonymous 0222* (SM); Yuexi County, 1960, *Sichuan Pharma. Exped. 26601* (NAS); Zhaojue County, Bi’er Town, alt. 2470 m, 19 June 1979, *Zhaojue Exped. 605* (SM); Zhaojue County, E’er Town, alt. 1990 m, 2 June 1979, *Zhaojue Exped. 0452* (SM); Zhaojue County, Wupo Township, alt. 2100 m, 29 June 1976, *Sichuan Plant Exped. 12749* (CDBI, NAS); 1960, *Sichuan Pharma. Exped. 27871* (NAS). • **Xizang Province**, Bomê County, alt. 2700 m, 29 June 1973, *Tibet Exped. 770* (HNWP); Bomê County, Gu Township, 29°55'N, 95°30'E, alt. 3080 m, 19 June 1965, *J.S. Ying & D.Y. Hong 650295* (PE); Bomê County, Zhamo Town, alt. 2800 m, 13 July 1965, *Y.T. Chang & K.Y. Lang 597* (PE); Mainling County, Hongwei Forest Farm, alt. 3200–3300 m, 29 July 1975, *Tibet Exped. 750998* (HNWP, KUN); Nyingchi City, nearby Northern Yarlung Zangbo River Valley, alt. 3100 m, 30 May 1973, *Tibet Exped. 141* (HNWP). • **Yunnan Province**, Anning City, Bijia Mountain, alt. 2100 m, 15 July 1965, *C.Y. Wu 292* (KUN, WUK); Binchuan County, alt. 2800 m, 20 July 1933, *H.T. Tsai 53699* (KUN, NAS, PE); Binchuan County, Mount Jizu, 10 August 1920, *Anonymous 789* (PEY); Binchuan County, Mount Jizu, Jinding Temple, alt. 2700 m, 9 September 1984, *Z.H. Pan et al. 84–33* (NAS); Binchuan County, Mount Jizu, nearby Zhusheng Temple, alt. 2300 m, 10 September 1984, *Z.H. Pan et al. 84–50* (NAS); Dali City, Eastern slope of Cangshan Mountain, 22 June 1929, *Ching 22992* (KUN, PE); Dêqên County, Huan-fu-ping, alt. 3000 m, September 1935, *C.W. Wang 69305* (LBG, NAS, PE, WUK); Dêqên County, Huan-fu-ping, 27 September 1959, *K.M. Feng 23736* (KUN); Heqing County, Lianping, Nanqingtou, 8 August 1929, *Ching 23581* (PE); Heqing County, Lianping, Zhaobi Mountain, alt. 2800 m, 4 August 1929, *Ching 24074* (KUN, PE); Heqing County, San tchang kiou, alt. 2500 m, 6 August 1885, *M. Delavay 2030* (K, NY, P); Heqing County, Swallow Cave, Ma’er Mountain, alt. 2800 m, 31 July 1963, *NW Yunnan Jinsha River Exped. 4744* (PE); Heqing County, 1906, *F. Ducloux 4245* (P); Huize County, *O. Schoch 217* (K, US); Huize County, Tianba Village, alt. 2300 m, 5 September 1986, *Z.H. Pan et al. 86005* (CDBI, NAS); Huize County, Tianba Township, Fendou Village, 25°56'31.74"N, 103°30'41.62"E, alt. 2292 m, 16 June 2025, *H.M. Li & J.W. Zhu 3002* (NAS); Huize County, Tianba Township, Hongyan Village, 25°53'0.50"N, 103°26'26.21"E, alt. 2078 m, 16 June 2025, *H.M. Li & J.W. Zhu 3003* (NAS); Kunming City, 6 July 1938, *F.N. 10685* (PEY); Kunming City, Dongchuan District, Yinmin Town, alt. 2290 m, 20 June 1985, *S.B. Lan 433* (PE); Kunming City, Xishan District, Tuanjie Township, alt. 2017 m, 25°05'08.30"N, 102°27'42.74"E, 17 June 2025, *H.M. Li & J.W. Zhu 3004* (NAS); Lijiang City, alt. 3048 m, July 1914, *G. Forrest 12701* (E); Lijiang City, Wenbi Mountain, alt. 2600 m, 9 August 1962, *Shangri-La Exped. 647* (PE); Lijiang City, Wenfeng Temple, alt. 2800 m, 11 July 1935, *C.W. Wang 71399* (KUN, PE, WUK); Lijiang City, 26 October 1939, *Ching 21949* (KUN, PE); Lijiang City, western slope of Lijiang Alpine Botanic Garden, 26°59'58"N, 100°11'39"E, alt. 2798.8 m, 3 September 2024, *X.D. Ma 310* (NAS); Shangri-La City, Haba Snow Mountain, upper Longwangbian, alt. 2700 m, 2 September 1962, *Shangri-La Exped. 1864* (PE); Shangri-La City, Haba Snow Mountain, Haba Village, 27°22'48"N, 100°08'03"E, alt. 2731.49 m, 4 September 2024, *X.D. Ma 315* (NAS); Shangri-La City, road to Haba Shan, Sanba, 27°30'51"N, 100°02'05"E, alt. 2724 m, 15 June 1994, *ACE266* (E, K); Shangri-La City, *T. Zhang et al. 11CS3313* (KUN); Shilin County, Guishan, 24°38'56.00"N, 103°35'51.2"E, alt. 2250 m, 29 October 2006, *Y.M. Shui et al. 65492* (KUN); Songming County, Guodong Village, *B.Y. Qiu 54954* (KUN, PE); Songming County, in the vicinity of Longtan,25°28'N, 102°46'E, alt. 2100 m, 27 July 1984, *1984 Sino-Amer. Bot. Exped. 1317* (US); Weishan County, Weibaoshan, 1933, *Y. Tsiang 11965* (KUN, NAS, PE); Weixi County, Kangpu, alt. 1932 m, July 1935, *C.W. Wang 64134* (KUN, NAS, PE); Weixi County, Pantiange, *Anonymous 61–0130* (NAS); Weixi County, Yeh-Chih, alt. 3400 m, August 1935, *C.W. Wang 68296* (NAS, PE); Xundian County, Hai-Chia, alt. 2700 m, 16 November 1940, *Y.P. Chang 0963* (PE); Zhaotong City, Qiaojia, Da Village, Jibaka, 27°12'35.58"N, 103°06'40.26"E, alt. 2887 m, 25 June 2012, *Z.X. Ren, W. Jiang, M.Y. Zhang WH–2012–0456* (KUN); Zhaotong City, Qiaojia, Dakua Mountain, 25°11'53.6"N, 103°07'24.1"E, alt. 2794 m, 23 August 2007, *Z.X. Ren, L.N. Dong, P.H. Huang SCSB-W–291* (KUN); Zhaotong City, Qiaojia, Zhongchang, 27°01'51.77"N, 103°00'06.64"E, alt. 2730 m, 22 August 2007, *S.D. Zhang et al. SCSB-W–327* (KUN); *Anonymous 12053* (PEY); *Chen 811* (NAS); *E.E. Maire 508* (E); *E.E. Maire 1253* (E); *R.N. 10685* (PEY); *H.T. Tsai 57553* (KUN, PE); 20 July 1903, *F. Ducloux 2237* (P); 1938, *W.K. Hsia 57553* (PE); 25 July 1968, *F. Ducloux 5939* (P); 17 June 1973, *B.X. Sun et al. 687* (KUN); 9 August 1979, *Anonymous 610129* (NAS).

##### Notes.

1) *Sanicula astrantiifolia* was described on the basis of three collections, namely *E.E. Maire s.n.*, *S. Ten 287* and *O. Schoch 217.* We have traced one specimen of the first collection at P (Fig. 1B) and two specimens of the last collection at K and US (Fig. 1C, D). All these specimens match the protologue well and are syntypes as Wolff (1930) did not designate a type for *S. astrantiifolia* (McNeill 2014). According to ICN Art. 9.17 (Turland et al. 2018), Pimenov (2017) designated the P sheet as lectotype of this species; here we accept his treatment.

2) Sheh and Phillippe (2005) categorized *Sanicula potaninii* Bobrov as a synonym of *S. astrantiifolia* without providing sufficient rationale. Subsequently, Pimenov (2017) also classified *S. potaninii* as a synonym of *S. serrata* H. Wolff. However, our examination of living specimens and critical herbarium collections indicate that *S. potaninii* can be distinctly differentiated from *S. astrantiifolia* and *S. serrata* based on its habit and leaf morphology. Furthermore, *S. potaninii* shows a close affinity with S. serrata var. uncinata Shan & Constance, particularly in inflorescence and leaf characteristics. A thorough discussion regarding the taxonomic status of *S. potaninii* will be addressed in a separate analysis.

## Supplementary Material

XML Treatment for Sanicula rugulosa

## ﻿Additional information

### Use of AI

No use of AI was reported.

### Author ORCIDs

Hui-Min Li https://orcid.org/0000-0001-6660-7620

Jun-Wen Zhu https://orcid.org/0009-0000-5554-445X

Xu-Dong Ma https://orcid.org/0009-0006-6303-0006

Si-Rong Yi https://orcid.org/0000-0002-3883-6175

Chun-Feng Song https://orcid.org/0000-0003-2077-0486

### Data availability

All of the data that support the findings of this study are available in the main text or Supplementary Information.

## Supplementary materials

Supplementary material 1 Individual-level d1/d2 ratio values for each population Data type xlsx Explanation note Corresponding to the boxplots in Fig. 7A, B. https://binary.pensoft.net/file/1414464This dataset is made available under the Open Database License (http://opendatacommons.org/licenses/odbl/1.0/). The Open Database License (ODbL) is a license agreement intended to allow users to freely share, modify, and use this Dataset while maintaining this same freedom for others, provided that the original source and author(s) are credited.Hui-Min Li, Jun-Wen Zhu, Xu-Dong Ma, Si-Rong Yi, Chun-Feng Song

Supplementary material 2 Individual-level r1/r2 ratio values for each population Data type xlsx Explanation note Corresponding to the boxplots in Fig. 7C, D. https://binary.pensoft.net/file/1414465This dataset is made available under the Open Database License (http://opendatacommons.org/licenses/odbl/1.0/). The Open Database License (ODbL) is a license agreement intended to allow users to freely share, modify, and use this Dataset while maintaining this same freedom for others, provided that the original source and author(s) are credited.Hui-Min Li, Jun-Wen Zhu, Xu-Dong Ma, Si-Rong Yi, Chun-Feng Song
